# What makes the psychosis ‘clinical high risk’ state risky: psychosis itself or the co-presence of a non-psychotic disorder?

**DOI:** 10.1017/S204579602100041X

**Published:** 2021-07-06

**Authors:** Laila Hasmi, Lotta-Katrin Pries, Margreet ten Have, Ron de Graaf, Saskia van Dorsselaer, Maarten Bak, Gunter Kenis, Alexander Richards, Bochao D. Lin, Michael C. O'Donovan, Jurjen J. Luykx, Bart P.F. Rutten, Sinan Guloksuz, Jim van Os

**Affiliations:** 1 Department of Psychiatry and Neuropsychology, School for Mental Health and Neuroscience, Maastricht University Medical Centre, Maastricht, the Netherlands; 2 Department of Epidemiology, Netherlands Institute of Mental Health and Addiction, Utrecht, The Netherlands; 3 FACT, Mondriaan Mental Health, Maastricht, Netherlands; 4 MRC Centre for Neuropsychiatric Genetics & Genomics, Division of Psychological Medicine & Clinical Neurosciences, Cardiff University, Cardiff, United Kingdom; 5 Department of Translational Neuroscience, UMC Utrecht Brain Center, University Medical Center Utrecht, Utrecht University, Utrecht, The Netherlands; 6 Department of Neurology, Brain Centre Rudolf Magnus, University Medical Centre Utrecht, Utrecht, the Netherlands; 7 GGNet Mental Health, Apeldoorn, The Netherlands; 8 Department of Psychiatry, UMC Utrecht Brain Center, University Medical Center Utrecht, Utrecht University, Utrecht, The Netherlands; 9 Department of Psychiatry, Yale University School of Medicine, New Haven, CT, USA; 10 Department of Psychosis Studies, Institute of Psychiatry, Psychology & Neuroscience, King's College London, London, UK

**Keywords:** Epidemiology, prevention, psychosis, risk

## Abstract

**Aims:**

Although attenuated psychotic symptoms in the psychosis clinical high-risk state (CHR-P) almost always occur in the context of a non-psychotic disorder (NPD), NPD is considered an undesired ‘comorbidity’ epiphenomenon rather than an integral part of CHR-P itself. Prospective work, however, indicates that much more of the clinical psychosis incidence is attributable to prior mood and drug use disorders than to psychosis clinical high-risk states *per se*. In order to examine this conundrum, we analysed to what degree the ‘risk’ in CHR-P is indexed by co-present NPD rather than attenuated psychosis *per se*.

**Methods:**

We examined the incidence of early psychotic experiences (PE) with and without NPD (mood disorders, anxiety disorders, alcohol/drug use disorders), in a prospective general population cohort (*n* = 6123 at risk of incident PE at baseline). Four interview waves were conducted between 2007 and 2018 (NEMESIS-2). The incidence of PE, alone (PE-only) or with NPD (PE + NPD) was calculated, as were differential associations with schizophrenia polygenic risk score (PRS-Sz), environmental, demographical, clinical and cognitive factors.

**Results:**

The incidence of PE + NPD (0.37%) was lower than the incidence of PE-only (1.04%), representing around a third of the total yearly incidence of PE. Incident PE + NPD was, in comparison with PE-only, differentially characterised by poor functioning, environmental risks, PRS-Sz, positive family history, prescription of antipsychotic medication and (mental) health service use.

**Conclusions:**

The risk in ‘clinical high risk’ states is mediated not by attenuated psychosis *per se* but specifically the combination of attenuated psychosis and NPD. CHR-P/APS research should be reconceptualised from a focus on attenuated psychotic symptoms with exclusion of non-psychotic DSM-disorders, as the ‘pure' representation of a supposedly homotypic psychosis risk state, towards a focus on poor-outcome NPDs, characterised by a degree of psychosis admixture, on the pathway to psychotic disorder outcomes.

## Introduction

There is considerable interest in the psychosis clinical High-Risk state (CHR-P) as a paradigm to identify the risk factors, mechanisms and early intervention potential associated with the onset of psychotic disorder (Fusar-Poli *et al*., 2020). CHR-P is defined mainly by the presence of attenuated psychotic symptoms which, accordingly, represent the central part of the definition of attenuated psychosis syndrome (APS) in (the appendix of) DSM-5 (Tsuang *et al*., 2013).

Although some have proposed that the CHR-P/APS construct may be valid (Woods *et al*., 2009; Salazar de Pablo *et al*., 2020), others point to epistemic, conceptual and methodological limitations surrounding different aspects of CHR-P research (van Os and Guloksuz, 2017; Ajnakina *et al*., 2018; Moritz *et al*., 2019). Clarification of these issues is urgently required given increasingly large-scale research projects based on the CHR-P paradigm.

One major validity issue is related to non-psychotic ‘comorbidity’ in CHR-P research. Non-psychotic ‘comorbidity’ does not form part of the CHR-P/APS construct. Rather, it is treated as an exclusion factor in the criteria for APS and CHR-P through formulations like ‘*attenuated psychotic symptoms are not explained better by another DSM disorder*’ (Salazar de Pablo *et al*., 2020). Nevertheless, the samples collected in the context of CHR-P research invariably show that the vast majority of individuals (around 80%) have a diagnosis of non-psychotic disorder (NPD). A recent systematic review including 56 studies showed that 49% presented with comorbid depressive disorders, 22% with bipolar disorder, 38% with anxiety disorders and 20% with substance use disorders (sum of percentages is greater than 100 due to comorbidity) (Salazar de Pablo *et al*., 2020).

This state of affairs represents an unacceptable conundrum: according to definitions, CHR-P/APS can only include attenuated psychotic symptoms that are not ‘better explained’ by another DSM-disorder – yet the vast majority of individuals meeting CHR-P/APS criteria present with another DSM-disorder. The likely explanation for this apparent paradox is mis-specification of the CHR-P/APS construct itself. Thus, the co-presence of NPD in CHR-P/APS samples may not be an ‘unwelcome’ epiphenomenon but rather represent an integral part of the CHR-P/APS construct itself. NPD may be what mediates the ‘risk’ in the concept of CHR-P and represent the mechanism for the relatively poor outcome over time associated with the CHR-P/APS state, whether or not ‘transition’ took place (Lin *et al*., 2011). While this explanation initially may not seem straightforward, it is from the perspective of population-based sampling. Research shows that individuals with mental health difficulties typically experience multiple diagnoses over the life course, often beginning with NPDs (Plana-Ripoll *et al*., 2019), whereas the artificially ‘pure’ CHR-P samples advance a competing, but incorrect, notion that psychosis is somehow inherently homotypic and therefore inconsistent with transdiagnostic symptoms (van Os and Guloksuz, 2017; Raballo and Poletti, 2019). However, population-based research shows that a prior diagnosis of mood disorder, neurotic disorder, eating disorder and substance use disorder – or basically any diagnosis outside the schizophrenia spectrum increases the risk of a later diagnosis of schizophrenia 10–20 fold (Plana-Ripoll *et al*., 2019) or 5-fold (Weiser *et al*., 2001). Indeed, recent prospective work showed that much more of the clinical psychosis incidence is attributable to prior mood and drug use disorders than to psychosis clinical high-risk states (Guloksuz *et al*., 2020).

One of the weaknesses of CHR-P sampling frames is that they are based on non-epidemiological opportunity sampling of selected help-seeking individuals with a NPD, yielding sample-specific results that are neither representative (Ajnakina *et al*., 2018) nor generalisable (van Os and Guloksuz, 2017). Indeed, ‘transitions’ in these individuals largely arise as a function of risk enrichment strategies embedded in specific sampling procedures (Fusar-Poli *et al*., 2016), limiting their use as a model of the onset of psychosis in the general population. Population-based cohort studies, however, can address the dynamics of attenuated psychotic symptoms and the role of NPD over time in representative samples (van Os *et al*., 2021). These studies have shown that the co-presence of NPD, present in around 50% of individuals with attenuated psychotic symptoms (Van Os *et al*., 2000; Jeppesen *et al*., 2015), is a crucial distinguishing factor in relation to aetiological load, clinical relevance and outcome (Hanssen *et al*., 2005; Kaymaz *et al*., 2007; van Rossum *et al*., 2011; Wigman *et al*., 2012; Pries *et al*., 2018; Radhakrishnan *et al*., 2019). Also, attenuated psychotic symptoms in isolation are either not (Jones *et al*., 2016; van Os *et al*., 2020), negatively (Hatzimanolis *et al*., 2018) or weakly (Pain *et al*., 2018; Legge *et al*., 2019) associated with polygenic risk for schizophrenia (PRS-SZ), but tend to show progressively stronger association with PRS-SZ in combination with more environmental exposure, more affective comorbidity and more clinical relevance (Hatzimanolis *et al*., 2018; Guloksuz *et al*., 2019; Guloksuz *et al*., 2020). PE may be associated with subjective (Koyanagi *et al*., 2018) or objective cognitive alterations (Niarchou *et al*., 2013; Gur *et al*., 2014; Rossler *et al*., 2015); there is evidence, however, that the association with cognitive alterations is dependent on the degree of comorbid non-psychotic psychopathology (Reininghaus *et al*., 2019). Indeed, recent follow-back studies from representative incidence samples of psychotic disorder have shown that the origins of psychotic disorder can be traced to NPDs, the severest of which develop a degree of psychosis admixture over time (Cupo *et al*., 2021). Indeed, studies using prospective approaches show that nonpsychotic syndromes are frequently observed not just alongside psychotic experiences (PE)/symptoms, but even before them (Hafner *et al*., 1999; Shah *et al*., 2019).

Here, we investigated the clinical significance of the co-presence of NPD in first-onset PE. To this end we calculated, for the first time, the incidence rate of PE, alone (PE-only) and as a function of co-presence of NPD (PE + NPD - mood disorders, anxiety disorders and alcohol/drug use disorders) in a risk set of people without PE at baseline. We also analysed the differential impact of relevant clinical, aetiological, cognitive and demographic factors on the incidence of PE-alone and PE + NPD.

We hypothesised that a minority of people with incident PE would have co-presence of NPD, and that clinical, demographic, aetiological and cognitive factors, known to be associated with psychotic disorder, would display stronger and/or qualitatively different associations with the incident PE + NPD phenotype in comparison with the PE-only phenotype.

## Method

### Sample

The four waves of the Netherlands Mental Health Survey and Incidence Study-2 (NEMESIS-2) were used (*n* = 6646 at baseline or T0). NEMESIS-2 was conducted over the period 2007–2018 to study the prevalence, incidence, course, and consequences of mental disorders in a representative sample of the Dutch general population (for description see online Supplementary material).

#### Sample risk set

Individuals, with confirmed psychosis at baseline and therefore not at risk anymore of developing incident psychosis, were not included in the risk set. Thus, individuals with a diagnosis of any psychotic disorder according to the DSM-IV (*n* = 43) were excluded from analysis. Also, those who had PE present in the year before T0 were excluded (*n* = 480), leaving 6123 participants at T0 considered at risk of developing incident PE. Individuals who had PE in the past, but more than 1 year before baseline and not in the year before baseline, were considered at risk of developing new incident PE and included in the risk set. The 6123 individuals thus included in the risk set yielded 19 115 observations over T0-T3. Of the 6123 individuals at T0, 4930 remained at T1 after a mean follow-up of 3.02 years; 4314 remained at T2, after a mean follow-up of 6.01 years; and 3748 remained at T3, after a mean follow-up of 9.04 years.

A planned sensitivity analysis was carried out with a more restricted risk set, excluding all participants with any PE at baseline (*n* = 5565 at baseline, total sample *n* =  17 282).

Given the age range of the sample (18–65 years at baseline), another sensitivity analysis was conducted in order to examine if results would be similar when restricted to the sample aged 18–35 at baseline (*n* = 1439, 26%), the age range during which most psychosis onset takes place.

As the definition of non-psychotic diagnoses (NPD) included drug abuse and dependence, a further sensitivity analysis of the association with cannabis use was conducted excluding individuals with drug abuse or dependence from the definition of NPD.

Finally, for the measures split at the xth percentile (social functioning, digit span, childhood trauma, PRS), which may be considered arbitrary, we included sensitivity analyses with continuous scores.

### Assessment of NPDs

The following CIDI, version 3.0, non-psychotic diagnoses (NPD) were assessed, as described in the online Supplementary material: major depression, dysthymia, bipolar disorder, panic disorder, agoraphobia, social phobia, specific phobia, GAD, alcohol abuse and dependence, drug abuse and dependence. The analyses thus focused on people who developed incident PE over the period of observation, either alone (PE-only) or in the co-presence of NPD (PE + NPD).

### Assessment of PE

In NEMESIS-2, a psychosis add-on instrument based on the G section of previous CIDI-versions was included. This add-on instrument consists of 20 psychotic symptoms corresponding to the symptoms assessed in a previous population survey in the Netherlands, NEMESIS, the precursor of NEMESIS-2 (Bijl *et al*., 1998; de Graaf *et al*., 2010). Detailed descriptions of the specific PE items can be found in previous work using NEMESIS (Smeets *et al*., 2013) and NEMESIS-2 (van Nierop *et al*., 2012) and are described in the online Supplementary material. PE was dichotomised consistent with previous work in NEMESIS and NEMESIS-2 (van Rossum *et al*., 2011; Pries *et al*., 2018; Radhakrishnan *et al*., 2019). Presence of delusions was defined as having at least one delusion endorsed and presence of hallucinations was similarly defined (online Supplementary material).

### Exposure variables

We examined 17 exposures associated with psychotic disorder. Dichotomous measures of exposure were created at the 75th percentile, unless a previous publication had used another cut-off in which case this was used for reasons of consistency (see below).

#### Working memory performance

The digit-span task, subtest of the Wechsler Adult Intelligence Scale (WAIS-III) (Wechsler, 1997), was performed by participants at T1 and T3. The digit-span task was split into two sections, a forward (six items) and backward (six items) task condition. The sum score at T1 and T3 was computed, and the average of these two values was considered as a person-level indicator of cognitive ability for all waves. In the analysis, a dichotomised variable was used, with cut-off at the 75th percentile, the highest value indicating poorer performance.

#### Jumping to conclusion bias (JTC bias)

The presence or absence of a JTC bias was assessed at T2, utilising the beads task (online Supplementary material) and used as a person-level, time-invariant dichotomous variable in the analyses.

#### Childhood adversity

Childhood adversity was assessed at T0 using a questionnaire based on the NEMESIS trauma questionnaire (de Graaf *et al*., 2010), and used as a dichotomous variable in the analyses (online Supplementary material).

#### Cannabis use

Cannabis use was assessed with the section substance use disorders of the CIDI at each interview wave and used as a dichotomous variable in the analyses (online Supplementary material).

#### Urbanicity

The degree of exposure to the urban environment until the age of 16 years, was assessed at T0 and used as a dichotomous variable in the analyses (online Supplementary material).

#### Family history

Family history was assessed as a person-level characteristic in two stages, as described previously (Radhakrishnan *et al*., 2019) and detailed in the online Supplementary material.

#### Hearing impairment

Hearing impairment was assessed during the face-to-face interview at all interview waves, by asking whether participants had experienced deafness or serious hearing impairment in the past 12 months. Ratings were yes (1) or no (0).

#### Social functioning

The evaluation of social functioning covered the past 4 weeks, and was assessed at each interview wave, applying a 2-item, 6-point subscale of the Medical Outcomes Study Short-form Health Survey (MOS SF-36) (Stewart *et al*., 1988; Ware and Sherbourne, 1992), with a Cronbach's alpha of 0.78. Impaired social functioning includes issues in one's normal social activities as a result of somatic or emotional troubles. It was used as a binary variable in the analysis, dichotomised around the 75th percentile.

#### Service use for mental problems

Use of any form of care or specific mental health dare, as well as use of antipsychotic medication, was assessed at each interview wave (online Supplementary material).

#### Perceived status gap

The *perceived status gap* is a dichotomous variable indicating the difference between the subjective desired and actual social status (online Supplementary material).

#### Adult stressful life events

Based on the ‘Brugha Life events section’ (Brugha *et al*., 1985), participants were asked at each interview whether they experienced one of nine life events within the last 12 months. Examples of items are serious sickness, death of family member or close friend, and serious financial problems. A dichotomous exposure was created around at least one life event in the last year.

#### Polygenic risk score for schizophrenia

The PRS-SZ were created from best-estimate genotypes at six different *p*-thresholds (0.5, 0.1, 0.05, 5×10^−3^, 5×10^−5^, 5×10^−8^), as described in the online Supplementary material. For our primary analyses, we used the *p*-threshold of <0.05, as this threshold explained most variation in liability in the Psychiatric Genomics Consortium analysis (Schizophrenia Working Group of the Psychiatric Genomics Consortium, 2014) and was shown to perform well for the current phenotype of SF-36 mental health (Pries *et al*., 2019). Further details on the genotyping procedure and polygenic risk scores calculation, as described previously (Pries *et al*., 2018; Guloksuz *et al*., 2020), are provided in the supplement. Consistent with previous analyses, statistical analyses with PRS-SZ were adjusted for three principal components (Pries *et al*., 2020).

Material for DNA analysis of sufficient quality, and hence for polygenic risk scores calculation, was available for 3104 individuals (47%) at T0 (online Supplementary material). Excluding individuals who at interview has been assessed as member of an ethnic minority (online Supplementary material), given lack of generalisability of polygenic risk scores to this group, and individuals diagnosed with a psychotic disorder, left 3037 for PRS calculation, of whom 2836 remained in the risk set for incidence analysis as defined below. These 2836 with polygenic risk scores yielded 9737 observations over the four interview waves. Values for important and time-varying clinical, environmental, cognitive and demographic variables were very similar in a comparison between the 9737 observations in the subsample with polygenic risk scores data available and the 10 238 observations in the risk set with missing polygenic risk scores data (online Supplementary Table 2).

#### Demographic factors

Demographic variables included were sex (0 = male, 1 = female), age in years and dichotomous ethnic minority status (Moroccan, Turkish, Surinamese, Antillean, Indonesian or other non-Western ethnic group). Age was analysed as a dichotomous variable, defining a younger age group encompassing the range most at risk of onset of psychotic disorder (18 to 35 years, 24% at baseline) versus the older group.

### Analysis

#### PE-only and PE + NPD groups

Two outcomes were defined to test the hypotheses regarding PE in relation to the context of NPD. The first was defined as PE without NPD, the second as PE in the co-presence of NPD. For each group we established (i) the incidence and (ii) associations with cognitive, clinical, demographic and aetiological variables, expressed as hazard ratio's (HR) and their 95% confidence intervals (CI) from Cox proportional hazards regression.

#### Analysis of incidence and predictors of incidence

The STSET command was used to obtain the format of survival data in Stata, guiding treatment of time-varying and fixed independent variables in the analyses. Incidence was calculated using failures in single-failure per subject data (i.e. one single event was defined as failure, with a single record per subject), divided by the number of person-years. Incidence was calculated using the Stata STSUM routine. Cox regression was done using the Stata STCOX routine. The proportional-hazards assumption was tested with the Stata ESTAT PHTEST routine, which detected no violations.

## Results

### Sample characteristics

Mean age of the 6123 participants in the risk set at T0 was 44.4 years (s.d. = 12.5, range 18–68), 55% were female. More than a third (37%) had a higher professional education or university degree, 32% had completed up to higher secondary education, 27% up to lower secondary education and 5% had completed primary education only; 74% were married or cohabiting; 20% lived alone; 70% were in paid employment; 7% pertained to an ethnic minority group.

### Incidence of PE-only and PE + NPD combinations

The incidence of PE-only was 1.04% (Table 1). The incidence of NPD and PE combined was 0.37%, or around 25% of the total PE incidence [(0.37/ (1.04 + 0.37)] (Table 1).

**Table 1.** Incidence of PE, either alone or co-present with NPDs

Incident experience	Participants	Time at risk (years) ^a^	Number of incident cases	Incidence %
PE-only	4930	37 898.1	395	1.04
PE + NPD	4930	38 721.3	142	0.37

PE: psychotic experiences; NPD: non-psychotic disorder.

a The total number of person-years, derived from the sum of persons and the length of their individual follow-ups, in years.

### PE-only and PE + NPD associations with clinical, demographic, cognitive and aetiological factors

**Fig. 1.**
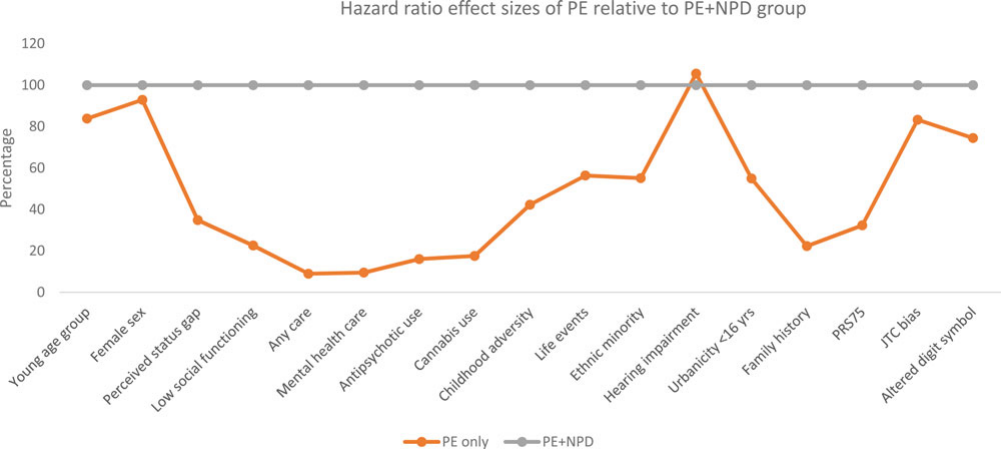
Hazard ratio (HR) effect sizes of binary clinical, demographic, aetiological and cognitive factors in PE-only group relative to effect sizes of PE + NPD group (set at 100%, grey line). Young age group: aged 18–35 years; Perceived status gap: difference between actual and desired social position; Low social functioning: SF36 social functioning 75th percentile cut-off; Any care: any informal, medical or mental health care for mental problems or addiction; Cannabis use: once per week or more in the period of most frequent use; Childhood adversity: 80th percentile cut-off continuous adversity score before age 16 years; Life events: at least one life event in the last year; Minority: Moroccan, Turkish, Surinamese, Antillean, Indonesian or other non-western ethnic group; Hearing impairment: T0 deafness or serious hearing impairment in the past 12 months; Urbanicity: 2 highest levels of 5-level urbanicity classification before age 16 years; Family history: family history mental disorder; PRS_75_: schizophrenia polygenic risk score 75th percentile cut-off; JTC: beads task decision 2 or less beads; Altered digit symbol: cut-off 75th percentile continuous score.

The distribution of clinical, demographic, cognitive and aetiological factors tended to be different across the PE-only and PE + NPD groups, in comparison with the remainder of the sample, for most variables (Table 2). online Supplementary Table 3 displays the pattern of time-varying and time-constant exposures for the PE-only and PE + NPD incidence analysis. Online Supplementary Table 4 details the person-years, failures and incidence rates of PE-only and PE + NPD as a function of the various binary demographic, clinical, aetiological and cognitive risk factors. Hazard ratios are presented in Table 3 and the pattern of results is summarised in Fig. 1.

**Table 2.** Distribution of risk factors (proportions) as a function of PE, either alone or in combination with NPD across T0, T1, T2 and T3 repeated observations (6123 individuals yielding 19 115 observations)

Binary exposure	No PE (*n* = 18 476) (proportion)	PE-only (*n* = 462) (proportion)	PE + NPD (*n* = 177) (proportion)	Total (*n* = 19 115) (proportion)	Total exposed (*n*)
Young age group	0.16	0.16	0.18	0.16	3045
Female sex	0.54	0.60	0.66	0.55	10 458
Perceived status gap	0.18	0.27	0.53	0.18	3487
Low social functioning	0.32	0.42	0.77	0.33	6217
Any care	0.11	0.15	0.64	0.12	2233
Mental health care	0.06	0.07	0.42	0.06	1193
Antipsychotic use	0	0.01	0.06	0.004	74
Cannabis use	0.02	0.01	0.04	0.02	348
Childhood adversity	0.18	0.26	0.49	0.18	3487
Life events	0.47	0.55	0.67	0.47	8980
Ethnic minority	0.07	0.08	0.16	0.07	1363
Hearing impairment	0.03	0.06	0.06	0.03	549
Urbanicity <16 years	0.39	0.33	0.50	0.39	7377
Family history	0.58	0.72	0.92	0.59	11 217
PRS_75_	0.25	0.22	0.42	0.25	2331
JTC bias	0.51	0.54	0.61	0.51	8481
Altered digit symbol	0.28	0.39	0.50	0.29	4144

Young age group: aged 18–35 years; Perceived status gap: difference between actual and desired social position; Low social functioning: SF36 social functioning 75th percentile cut-off; Any care: any informal, medical or mental health care for mental problems or addiction; Cannabis use: once per week or more in the period of most frequent use; Childhood adversity: 80th percentile cut-off continuous adversity score before age 16 years; Life events: at least one life event in the last year; Minority: Moroccan, Turkish, Surinamese, Antillean, Indonesian or other non-western ethnic group; Hearing impairment: T0 deafness or serious hearing impairment in the past 12 months; Urbanicity: 2 highest levels of 5-level urbanicity classification before age 16 years; Family history: family history mental disorder; PRS_75_: schizophrenia polygenic risk score 75th percentile cut-off; JTC: beads task decision 2 or less beads; Altered digit symbol: cut-off 75th percentile continuous score.

**Table 3.** Differential associations of incident PE, alone (PE-only) and in the context of NPD (PE + NPD), with demographic, clinical, aetiological and cognitive factors

Binary exposure	PE-only				PE + NPD			
	HR^a^	95% CI		*P*	HR	95% CI		*p*
Young age group	1.54	1.18	2.01	0.001	1.84	1.21	2.78	0.004
Female sex	1.27	1.04	1.56	0.019	1.37	0.98	1.93	0.069
Perceived status gap	1.66	1.33	2.08	0.000	4.77^a^	3.43	6.64	0.000
Low social functioning	1.40	1.15	1.71	0.001	6.21^a^	4.22	9.14	0.000
Any care	1.24	0.94	1.65	0.124	13.87^a^	9.83	19.58	0.000
Mental health care	1.03	0.70	1.52	0.886	10.79^a^	7.73	15.05	0.000
Antipsychotic use	2.73	1.02	7.31	0.046	16.98^a^	7.92	36.39	0.000
Cannabis use	1.82	0.58	5.67	0.302	10.36^b^	4.23	25.35	0.000
Childhood adversity	1.66	1.32	2.08	0.000	3.92^a^	2.82	5.46	0.000
Life events	1.33	1.09	1.62	0.005	2.35^a^	1.66	3.34	0.000
Ethnic minority	1.29	0.90	1.84	0.166	2.33^b^	1.45	3.75	0.000
Hearing impairment	2.07	1.34	3.18	0.001	1.96	0.96	4.00	0.065
Urbanicity <16 years	0.85	0.69	1.04	0.113	1.54^a^	1.11	2.14	0.010
Family history	1.91	1.54	2.37	0.000	8.57^a^	4.74	15.48	0.000
PRS_75_	0.80	0.56	1.13	0.204	2.47^a^	1.48	4.10	0.000
JTC bias	1.08	0.88	1.33	0.461	1.30	0.92	1.84	0.144
Altered digit symbol	1.56	1.24	1.97	0.000	2.10	1.41	3.12	0.000

HR = hazard ratio, 95% CI = 95% confidence interval. Young age group: aged 18–35 years; Perceived status gap: difference between actual and desired social position; Low social functioning: SF36 social functioning 75th percentile cut-off; Any care: any informal, medical or mental health care for mental problems or addiction; Cannabis use: once per week or more in the period of most frequent use; Childhood adversity: 80th percentile cut-off continuous adversity score before age 16 years; Life events: at least one life event in the last year; Minority: Moroccan, Turkish, Surinamese, Antillean, Indonesian or other non-western ethnic group; Hearing impairment: T0 deafness or serious hearing impairment in the past 12 months; Urbanicity: 2 highest levels of 5-level urbanicity classification before age 16 years; Family history: family history mental disorder; PRS_75_: schizophrenia polygenic risk score 75th percentile cut-off; JTC: beads task decision 2 or less beads; Altered digit symbol: cut-off 75th percentile continuous score.

a HR significantly greater in PE + NPD group compared to PE-only group, based on non-overlapping confidence intervals.

b HR significant in PE + NPD group but not in PE-only group.

The pattern of results, as displayed in Table 3 and Fig. 1, was that most hazard ratio effect sizes were much higher for PE + NPD as compared to PE-only. Perceived status gap, low social functioning, care use, antipsychotic use, childhood adversity, life events, PRS_75_, urbanicity and family history discriminated between PE-only and PE + NPD, as evidenced by non-overlapping confidence intervals of the hazard ratios. In addition, suggestive differences (large and significant hazard ratio in PE + NPD; small and non-significant hazard ratio in PE-only) were apparent for cannabis use and ethnic minority status. The suggestive differential association with cannabis use remained after excluding individuals with drug abuse/dependence from the definition of NPD (PE-only: odds ratio (OR) = 1.83, 95% CI: 0.59–5.69; PE + NPD: OR = 6.27, 95% CI: 1.99–19.76).

The sensitivity analysis with the more restricted risk set, excluding all participants with PE at baseline revealed results that were very similar to the results in Table 3, quantitatively and qualitatively (online Supplementary Table 5). The other sensitivity analysis restricted to individuals aged 18–35 at baseline (*n* = 1439, 26%) similarly revealed a very similar pattern of results (online Supplementary Fig. 2). Finally, for the measures split at the xth percentile (social functioning, digit span, childhood trauma, PRS) results for the continuous exposures yielded similar significant discrimination between PE-only and PE + NPD for social functioning, childhood trauma and PRS, and similar lack of significant discrimination between PE-only and PE + NPD for digit span (online Supplementary Table 6).

## Discussion

### Findings

We found that of the total incidence of PE (around 1.4%), approximately 25% involved a combined phenotype of PE + NPD. Compared to PE-only, the combined phenotype was differentially characterised by perceived status gap, low social functioning, care use, antipsychotic use, childhood adversity, life events, family history, urbanicity and PRS-SZ, with additional suggestive differences for cannabis use and ethnic minority status – a pattern of results that is close to findings reported for psychotic disorder (Howes and Murray, 2014). We suggest that differential associations between PE-only and PE + NPD with various risk factors confirms that PE-NPD is integral to transitions to psychosis, in line with recent prospective work showing that much more of the clinical psychosis incidence is attributable to prior mood and drug use disorders than to psychosis clinical high-risk states (Guloksuz *et al*., 2020). Other arguments are, first, clinical face validity, given that the finding of differential associations with poor functioning, use of mental health care and psychotropic medications represents actual clinical status differentiation, which is a necessary requirement for later poor outcome. Second, differential associations with greater aetiological loading (childhood adversity, cannabis use, polygenic score) are associated with poorer outcome of psychotic states (Van Os *et al*., 1998; Grech *et al*., 2005; Jonas *et al*., 2019). Third, prospective studies have shown greater risk of transition and other poorer outcomes of psychosis risk states for many of the factors differentiating between PE-only and PE + NPD in this study including childhood adversity and cannabis use (van Nierop *et al*., 2013; Trotta *et al*., 2015; Kraan *et al*., 2018), non-psychotic comorbidity (Hanssen *et al*., 2005; Rutigliano *et al*., 2016; Plana-Ripoll *et al*., 2019; Guloksuz *et al*., 2020) and social functioning (Kaymaz *et al*., 2012).

### Does PE-only reflect the risk for psychotic disorder?

The results indicate that 74% of the incidence of PE, namely the part not arising in combination with NPD is less likely to be of clinical relevance. Although the incidence of PE-only was not clinically neutral – as evidenced by some degree of association with most of the 17 factors under examination, these associations were (significantly) weaker as compared to PE + NPD and there was no association with health care use. These results concur with the previously documented suggestion that clinically relevant psychosis is an indicator of severity in the constellation with non-psychotic psychopathology, and should not be considered in isolation (van Os and Reininghaus, 2016; van Os and Guloksuz, 2017; Cupo *et al*., 2021).

### The role of PRS and cognitive alterations

PRS-SZ was not associated with PE-only in terms of either direction or significance of association, whereas the PE + NPD phenotype was significantly and more strongly associated. It could be argued that this represents a chance finding imputable to imposing an arbitrary cut-off on the continuous PRS-SZ. However, a sensitivity analysis with the continuous PRS-SZ quartile group score revealed similar results with additional evidence for dose–response association, supporting underlying causality (online Supplementary Table 7).

A plausible explanation for the differential associations with PRS-SZ may have to do with the nature of PRS-SZ. While generally interpreted as polygenic risk for a mental disorder, it can also be interpreted as polygenic risk for poor outcome of a manifestation of transdiagnostic psychopathology, which is conceptually different. PE are transient in 80% of cases whereas schizophrenia is transient in less than 20% of cases (Linscott and van Os, 2013). The differences in association with PRS-SZ thus may represent absence of association with relatively good outcome for the phenotype PE-only and a positive association with poor outcome for the phenotype PE + NPD. A recent study has reported a conceptually similar association between PRS-SZ and illness course in a clinical sample (Jonas *et al*., 2019).

Cognitive alterations, including the JTC task did not discriminate between PE-only and PE + NPD outcomes, in agreement with previous work (Niarchou *et al*., 2013; Gur *et al*., 2014; Rossler *et al*., 2015). The results are compatible with the suggestion that cognitive alterations may not represent the ‘core’ of the psychosis syndrome (van Os *et al*., 2020; Richards *et al*., 2020), but instead become associated with the current poor-outcome definition of psychotic disorders because they moderate, together with PRS-SZ, the outcome of early non-psychotic states (van Os *et al*., 2020). In the presence of high PRS-SZ, together with higher levels of environmental exposure, cognitive alterations may channel early states of non-psychotic psychopathology towards a poor outcome psychosis phenotype, whereas cognitive alterations in combination with low PRS-SZ may divert early psychopathology towards the more benign PE-only phenotype.

### Implications for CHR-P/APS

These findings suggest a significant, genetically rooted discriminative function between the PE-only phenotype with relatively benign outcome and the more poor outcome PE + NPD phenotype. In the same direction, our evidence supports further investigation, in prospective CHR-P settings, of the hypothesis that non-psychotic psychopathology represents a necessary factor in the ontogenesis of psychotic disorders, such that the pathway from psychosis risk to clinical psychosis outcome requires a non-psychotic intermediary state, interacting with multiple conditions including cognitive alteration, high PRS-SZ and environmental exposure. Indeed, a recent follow-back study of a representative incidence sample confirmed this supposition (Cupo *et al*., 2021). Therefore, CHR-P/APS research should be reconceptualised from a focus on attenuated psychotic symptoms with exclusion of DSM-disorders, as the ‘pure' representation of a supposedly homotypic psychosis risk state, towards a focus on poor-outcome NPDs, characterised by a degree of psychosis admixture, on the pathway to psychotic disorder outcomes (van Os and Reininghaus, 2016; Guloksuz *et al*., 2020; Cupo *et al*., 2021).

### Methodological issues

The results should be interpreted in the context of a number of limitations. First, although the sample was sizeable, many participants were past the period of greatest risk for psychosis, reducing power. However, contrary to what is often thought, mean age of onset of psychotic disorder in the general population, not selected for age cut-off or poor outcome-related specific diagnostic criteria, is around 30 years for men and 40 years for women, replicated across different studies of treated incidence over extended periods in geographically defined areas (Castle *et al*., 1993; Allardyce *et al*., 2007). The relatively high incidence of psychosis outcomes in the current study confirms the age-related incidence pattern of psychosis. Second, although most important time-varying measures were dynamically captured over time, others were not. Thus, the measures of cognition were only assessed twice and modelled as a person-level average, not permitting dynamic modelling of incidence states of cognitive alterations, similar to the incidence states of NPD and PE.

Third, PRS-SZ was available for less than 50% of the sample, however this is unlikely to have biased the results given similar distributions of dependent and independent variables as a function of PRS-SZ availability. Finally, some of the comparisons between the states of PE-only and PE + NPD suffered from low power. For example, there were substantial effect size differences in the association with cannabis use and PRS-SZ, however the statistical resolution of these differences was limited. Similarly, even though the sample was relatively large and the follow-up extensive, the incidence of the outcome of interest, PE + NPD, was not high, occurring in 177 participants in the risk set of whom 142 counted as incident. As a result, confidence intervals sometimes were wide, particularly for rarer exposures.

## Data Availability

The data on which this manuscript is based are not publicly available. However, data from NEMESIS-2 are available upon request. The Dutch ministry of health financed the data and the agreement is that these data can be used freely under certain restrictions and always under supervision of the principal investigator (PI) of the study. Thus, some access restrictions do apply to the data. The PI of the study is the last author of this paper and can at all times be contacted to request data. At any time, researchers can contact the PI of NEMESIS-2 and submit a research plan, describing its background, research questions, variables to be used in the analyses, and an outline of the analyses. If a request for data sharing is approved, a written agreement will be signed stating that the data will only be used for addressing the agreed research questions described and not for other purposes.

## Supplementary material

For supplementary material accompanying this paper visit http://dx.doi.org/10.1017/S204579602100041X. click here to view supplementary material
