# Supplementary material for: What makes the psychosis ‘clinical high risk’ state risky: psychosis itself or the co-presence of a non-psychotic disorder?
Source: Epidemiol Psychiatr Sci. 2021 Jul 6;30:e53. doi: 10.1017/S204579602100041X (PMC8264801; doi:10.1017/S204579602100041X)
Supplement: Supplementary file 1 [file S204579602100041Xsup001.docx]

SUPPLEMENTAL MATERIAL

Table of content

[METHODS 3](#_Toc73114274)

[NEMESIS-2 Sample 3](#_Toc73114275)

[Assessment of non-psychotic disorders 3](#_Toc73114276)

[Assessment of PE 3](#_Toc73114277)

[Working memory performance 4](#_Toc73114278)

[Jumping to conclusion bias 4](#_Toc73114279)

[Family history 5](#_Toc73114280)

[Childhood adversity 5](#_Toc73114281)

[Cannabis use 5](#_Toc73114282)

[Urbanicity 5](#_Toc73114283)

[Hearing impairment 5](#_Toc73114284)

[Social functioning 5](#_Toc73114285)

[Service use for mental problems 6](#_Toc73114286)

[Perceived status gap 6](#_Toc73114287)

[Adulthood stressful life events 6](#_Toc73114288)

[Genotyping procedures, quality control, imputation, and Polygenic Risk Score (PRS) calculation 6](#_Toc73114289)

[A. Target Genotype Data Processing 6](#_Toc73114290)

[Figure 1. Correlation of SNPs MAF from NEMESIS-2 dataset with the reference MAF. 7](#_Toc73114291)

[Figure 2. First 2PCs OF NEMESIS-2 samples with Hapmap 3 before exclusion of ethnic outliers 8](#_Toc73114292)

[Supplemental Table 1: The complex-LD regions (build GRCh37) removed for PCA analysis. 8](#_Toc73114293)

[B. Training schizophrenia GWAS summary statistic processing and calculating polygenic risk score for schizophrenia 9](#_Toc73114294)

[RESULTS 10](#_Toc73114295)

[Supplemental Table 2: Comparison of frequencies of time-varying and fixed variables in analysis as a function of missing PRS data in the risk set 10](#_Toc73114296)

[Supplemental Table 3: Pattern of time-varying and time-constant exposures for PE-only and PE+NPD incidence analysis 11](#_Toc73114297)

[Supplemental Table 4: Person-year incidence rates for PE-only and PE+NPD as a function of absence (0) or presence (1) of binary risk factors 12](#_Toc73114298)

[Supplemental Table 5: Sensitivity analysis of differential associations of incident Psychotic Experiences (PE), alone (PE-only) and in the context of Non-Psychotic Disorder (PE+NPD), with demographic, clinical, aetiological and cognitive factors, with more exclusive risk set (all individuals with PE at baseline excluded) 14](#_Toc73114299)

[Supplemental Table 6: Sensitivity analysis of differential associations of incident Psychotic Experiences (PE), alone (PE-only) and in the context of Non-Psychotic Disorder (PE+NPD), with continuous measures of exposure instead of dichotomization around the x^th^ percentile 16](#_Toc73114300)

[Sensitivity analysis in individuals aged 18-35 years at baseline 17](#_Toc73114301)

[DISCUSSION 18](#_Toc73114302)

[Supplemental Table 7. Sensitivity analyses of association between PRS, modelled as continuous quartile-group variable, and incidence of PE+NPD phenotype, showing dose-response relationship and significant linear trend. 18](#_Toc73114303)

[REFERENCES 19](#_Toc73114304)

# METHODS

## NEMESIS-2 Sample

The baseline data of NEMESIS-2 were collected from 2007 to 2009, follow-up continued until 2018. The study was approved by the Medical Ethics Review Committee for Institutions on Mental Health Care and written informed consent was collected from participants at each wave. To ensure representativeness of the sample in terms of age (between the ages of 18 and 65 at baseline), region, and population density, a multistage random sampling procedure was applied. Dutch illiteracy was an exclusion criterion. Non-clinician, trained interviewers applied the Composite International Diagnostic Interview (CIDI) version 3.0 (Alonso *et al.*, 2004; de Graaf *et al.*, 2008) and additional questionnaires during home visits. Details of NEMESIS-2 are provided elsewhere (de Graaf *et al.*, 2010; de Graaf *et al.*, 2012). The first wave (T0) enrolled 6,646 participants (response rate 65.1%; average interview duration: 95 minutes), who were followed up in 3 visits within 9 years: successive response rates at year 3 (T1), year 6 (T2), and year 9 (T3) were 80.4% (*n* = 5,303; excluding those who deceased; interview duration: 84 minutes), 87.8% (*n* = 4,618; interview duration: 83 minutes), and 86.8% (n = 4,007; interview duration: 102 minutes), respectively. Occurrence at baseline reflects lifetime occurrence; occurrence at T1 to T3 reflect approximately 3-year interval (T0-T1, T1–T2, and T2-T3) occurrence. Attrition between T_0_ and T_3_ was not significantly associated with any of the individual disorders at T_0_ after controlling for sociodemographic characteristics (de Graaf *et al.*, 2018).

## Assessment of non-psychotic disorders

The following CIDI, version 3.0, diagnoses were assessed: major depression, dysthymia, bipolar disorder, panic disorder, agoraphobia, social phobia, specific phobia, GAD, alcohol abuse and dependence, drug abuse and dependence. Non psychotic diagnoses were made according to the fourth version of the Diagnostic and Statistical Manual of Mental disorders (DSM-IV) at T0, T1, T2 and T3, using the Composite International Diagnostic Interview (CIDI) version 3.0 (World Health Organisation, 1990).

Validity (Reed *et al.*, 1998; Haro *et al.*, 2006) and test-retest reliability were determined, demonstrating good validity in providing diagnoses for almost all non-psychotic disorders and good to excellent kappa coefficients for most diagnostic sections (Wittchen, 1994).

For this study, the incidence of any NPD, thus defined between intake and each follow-up was assessed. As individuals with psychotic experiences at T0 were excluded from the risk set (see below), only people with NPD who for the first time ever developed psychotic experiences over the period of observation were studied. Participants with a diagnosis of schizophrenia, as assessed through CIDI interview and clinical follow-up interview detailed below, were excluded.

## Assessment of PE

As CIDI methodology to assess psychotic experiences in versions of CIDI 1 and CIDI 2 was not included in CIDI 3.0, a psychosis add-on instrument was constructed, based on the G-section of psychotic symptoms in CIDI versions 1 and 2. This part of the interview consisted of 20 psychotic experiences, each rated “yes,” “no,” “don’t know,” or “refuse,” over the lifetime period. At baseline, lifetime prevalence of PE was assessed and T0-T3 interviews focused on interval occurrence.

The 20 experiences corresponded to the symptoms assessed in previous population surveys in the Netherlands (Bijl *et al.*, 1998; de Graaf *et al.*, 2010); detailed descriptions of the specific PE items can be found in previous work using NEMESIS (Smeets *et al.*, 2013) and NEMESIS-2 (van Nierop *et al.*, 2012). Whenever a psychotic experience was endorsed, the subject was asked to state, on a 1 (rarely) to 4 (almost always) scale, how often this experience occurred (Frequency), how much it bothered them (Distress), and to what extent the experience had an influence on their daily professional and social activities (Impact). The sum scores for frequency and impact of psychotic experiences, as well as distress by psychotic experiences were calculated as the mean of the sum scores of these items across the 20 psychotic experiences. Psychotic experiences were considered secondary if all endorsed psychotic items were caused by use of drugs/alcohol or physical illness. Because clinical relevance of psychotic experiences may be difficult to diagnose by lay interviewers (Helzer *et al.*, 1985), and because the interviewers made no clinical judgment about participants’ answers, the reported experiences may be considered an extension of “self-report.”

A clinician performed follow-up telephone interview when participants reported a psychotic symptom to assess whether this symptom was a true PE using questions from the Structured Clinical Interview for DSM-IV. At baseline, a total of 1,081 participants (16.3%) endorsed at least one self-reported PE. Of these, 794 participated in clinical re-interview (73.5%), of whom 340 (42.8%) reported at least one clinician validated PE. At T1, 440 out of a total 5,303 (8.3%) participants reported that at least one self-reported PE had occurred since the previous interview. Of these, 367 (83.4%) participants were available for clinical re-interview, of whom 172 (46.9%) reported at least one clinician validated PE. At T2, 284 out of the total 4,618 (6.2%) participants reported at least one self-reported PE since the previous interview. Of these, 230 (81.0%) participants were available for clinical re-interview, of whom 135 (58.7%) reported at least one clinically validate PE. At T3, 222 out of the total 4,007 (5.5%) participants reported at least one self-reported PE since the previous interview. Of these, 207 (93.2%) participants were available for clinical re-interview, of which 77 (37.2%) reported at least one clinically validate PE.

PE were dichotomized consistent with previous work in NEMESIS and NEMESIS-2 (Radhakrishnan *et al.*, 2019; Pries *et al.*, 2018; van Rossum *et al.*, 2011). Presence of delusions was defined as having at least one delusion endorsed and presence of hallucinations was similarly defined.

Given similarities between (i) CIDI self-reported and clinician validated PE, (ii) CIDI ratings of primary and secondary psychotic experiences, and (iii) PE with and without distress, in terms of associations, predictive value and outcome (van der Steen *et al.*, 2019; van Nierop *et al.*, 2012; Bak *et al.*, 2003; Van Os *et al.*, 2000), CIDI self-reported PE, i.e. any rating of ‘yes’ for any of the 20 PE, were used in the analyses, thus increasing statistical power.

## Working memory performance

The digit-span task, subtest of the Wechsler Adult Intelligence Scale (WAIS-III)(Wechsler, 1997), was performed by participants at T1 and T3. The digit-span task was split into two sections, a forward (six items) and backward (six items) task condition. The sum score at T1 and T3 was computed, and the average of these two values was considered as a person-level indicator of cognitive ability for all waves. In the analysis, a dichotomized variable was used, with cut-off at the 75th percentile, the highest value indicating poorer performance.

## Jumping to conclusion bias

The “jumping to conclusions” bias (JTC bias) is a well-established finding in schizophrenia and is manifest, when performing the beads task, as a tendency to decide on the color of the jar after seeing only one or two beads. The presence or absence of a JTC bias was assessed at T2, utilizing the beads task. This is an experimental task aimed to measure individuals’ reasoning style in ambiguous situations (Phillips and Edwards, 1966). Participants were shown two jars containing red- and blue-coloured beads in opposite ratios. Similar to previous research, we used the more difficult version of the beads task with a colour ratio of 60:40 beads, to increase sensitivity to detect JTC bias in a general population sample (Reininghaus *et al.*, 2019). Following previous work, JTC bias was defined as making a decision based on two or less bead (Reininghaus *et al.*, 2019). JTC is typically considered more as a trait than a state (Moritz and Woodward, 2005; Menon *et al.*, 2002), therefore and in line with previous work (Rauschenberg *et al.*, 2020), the number of beads drawn at T2 was considered constant trough the four waves and used as a person-level, time-invariant variable to T0, T1 and T3.

## Family history

Family history was assessed as a person-level characteristic in two stages, as described previously (Radhakrishnan *et al.*, 2019). First, for participants who screened positive for the following CIDI psychiatric diagnoses, presence of the disorder in direct relatives was assessed at each interview wave: alcohol/drug abuse/dependence, depression/dysthymia, mania, and anxiety disorders (panic disorder, social phobia, agoraphobia, generalized anxiety disorder). More than 40% of the sample thus screened family history positive at any of the waves. Second, at T1, self-reported parental history of “severe anxiety or phobias”, “severe depression” and “delusions or hallucinations” were assessed in the entire sample: around 20% thus screened positive. Using these two sources of information, the proportion of the sample in which family history could be assessed (hereafter: ‘family history’) was 94%, as described previously (Radhakrishnan *et al.*, 2019).

## Childhood adversity

Childhood adversity was assessed at T0 using a questionnaire based on the NEMESIS trauma questionnaire (de Graaf *et al.*, 2010). Whenever a subject reported having experienced one of five types of childhood adversity before the age of 16 years [emotional neglect (not listened to, ignored, or unsupported), physical abuse (kicked, hit, bitten, or hurt with object or hot water), psychological abuse (yelled at, insulted, unjustly punished/treated, threatened, belittled, or blackmailed), peer victimization (bullying), and one time or more sexual abuse (any unwanted sexual experience)], they were asked to state how often it had occurred. The item ‘sexual abuse’ was rated on a scale of 1 (once) to 5 (very often), while all other items (namely, emotional neglect, physical abuse, psychological abuse, and peer victimization or bullying) were rated and on a scale of 1 (sometimes) to 4 (very often). Consistent with previous research, the childhood adversity score was dichotomized at the 80th percentile (van Os *et al.*, 2017; van Dam *et al.*, 2015; Heins *et al.*, 2011).

## Cannabis use

Cannabis use was assessed with the section substance use disorders of the CIDI at each interview wave. If subjects reported cannabis use, they were rated on frequency of use in the period of most frequent use on a scale of 1 (never) to 7 (every day). Consistent with previous work (Pries *et al.*, 2018; Radhakrishnan *et al.*, 2019), a binary variable (absent = “0” and present = “1”) was constructed by using the cut-off value of once per week or more in the period most frequent use.

## Urbanicity

The degree of exposure to the urban environment until the age of 16 years, assessed at T0, was defined at five levels based on the Dutch classification of residence topography or population density: (1) countryside (distances to facilities is larger), (2) village (<25 000 inhabitants), (3) small city (25 000–50 000 inhabitants), (4) medium city (50 000–100 000 inhabitants), (5) large city (>100 000 inhabitants). Consistent with previous work, the cut-off of at least > 50 000 inhabitants was used to define the binary variable of urban area (Guloksuz *et al.*, 2015).

## Hearing impairment

Hearing impairment was assessed during the face-to-face interview at all interview waves, by asking whether participants had experienced deafness or serious hearing impairment in the past 12 months. Ratings were yes (1) or no (0).

## Social functioning

The evaluation of social functioning covered the past 4 weeks, and was assessed at each interview wave, applying a 2-item, 6-point subscale of the Medical Outcomes Study Short-form Health Survey (MOS SF-36)(Ware and Sherbourne, 1992; Stewart *et al.*, 1988), with a Cronbach’s alpha of 0.78. Impaired social functioning includes issues in one’s normal social activities as a result of somatic or emotional troubles. It was used as a binary variable in the analysis, dichotomized around the 75^th^ percentile.

## Service use for mental problems

As described elsewhere (Ten Have *et al.*, 2013), any care received indicates at least one contact with informal care (alternative care providers, pastoral care, persons in one’s close social network, self-help groups, telephone help lines, online care), general medical care (general practitioners, company doctors, social work, home care or district nurses, physiotherapists or haptonomists, medical specialists or other professionals working within this care sector) or mental health care (psychiatrists, psychologists, psychotherapists, addiction care). Service use was measured based on the service use section of NEMESIS (Bijl and Ravelli, 2000) at each interview wave. A variable of receiving any mental health care was defined separately.

## Perceived status gap

The *perceived status gap* was assessed at T1, T2 and T3 using two questions. First, the MacArthur Scale of Subjective Social Status (Adler *et al.*, 2000) was used to rate subjective social status. In an easy pictorial format, it presents a "social ladder" with 10 levels and asks individuals to place an "X" on the rung on which they feel they stand. The second question was about a similar ladder, but this time with regard to the *desired* level of social status. The difference between the subjective desired and actual social status was used as independent variable in the analyses. The mean value of T1-T3 was used to replace missing values at T0. In the analyses, it was dichotomized around the perception of being more than one level below desired social status.

## Adulthood stressful life events

Based on the “Brugha Life events section”(Brugha *et al.*, 1985), participants were asked at each interview whether they experienced one of 9 life events within the last 12 months. Examples of items are serious sickness, death of family member or close friend, and serious financial problems. A dichotomous exposure was created around at least one life event in the last year.

# Genotyping procedures, quality control, imputation, and Polygenic Risk Score (PRS) calculation

## A. Target Genotype Data Processing

**Genotyping procedures and quality control steps before imputation.**

NEMESIS-2 samples were genotyped on an IPMCN chip (Institute of Psychological Medicine and Clinical Neurology, Cardiff University), which was custom-made for EUGEI (588,628 genotyped variants for 4,043 participants)(EUGEI investigators, 2014). Quality control (QC) was done using PLINK v1.9 (Purcell *et al.*, 2007) as follows**.** There were 3,861 samples matching with phenotypes. Single nucleotide polymorphisms (SNPs) and samples with call rates below 95% and 98%, respectively, were removed. A strict SNP QC only for subsequent sample QC steps was conducted. This involved a minor allele frequency (MAF) threshold > 10% and a Hardy-Weinberg equilibrium (HWE) *P*-value > 10^-5^, followed by linkage disequilibrium (LD) based SNP pruning (R^2^ < 0.5). This resulted in ~60K SNPs to assess sex errors (n=145), heterozygosity (F< 5xSD the standard deviation (SD), n= 73), and relatedness by pairwise identity by descent (IBD) values > 0.1 (n= 170). Genetic outliers (n=154) were identified by principal component analysis (PCA, see below). In total, 3,104 individuals passed these QC steps. After removing failing samples (n = 757), a regular SNP QC was performed (SNP call rate>95%, HWE p>1e-06, MAF> 0.16%; as the IPMCN chip contains many rare variants, half the SNPs would have been removed if we had applied MAF> 1%; therefore, we loosened MAF threshold to 0.16% =10/(2*sample size of 3,104)). Next, strand ambiguous SNPs and duplicate SNPs were removed, resulting in a total of 298,104 genotyped variants.

1. **Imputation on Michigan server.**

The QC-ed dataset was chunked by chromosome, and then converted into *.VCF files. The Michigan server was used for imputation with the following settings: reference panel as HRC R1.1 2016; phasing as Eagle v2.3; population as European; model as QC & imputation. The imputation resulted in 47,101,073 single nucleotide polymorphisms (SNPs). The general imputation quality is shown in **Figure 1**.

## Figure 1. Correlation of SNPs MAF from NEMESIS-2 dataset with the reference MAF.


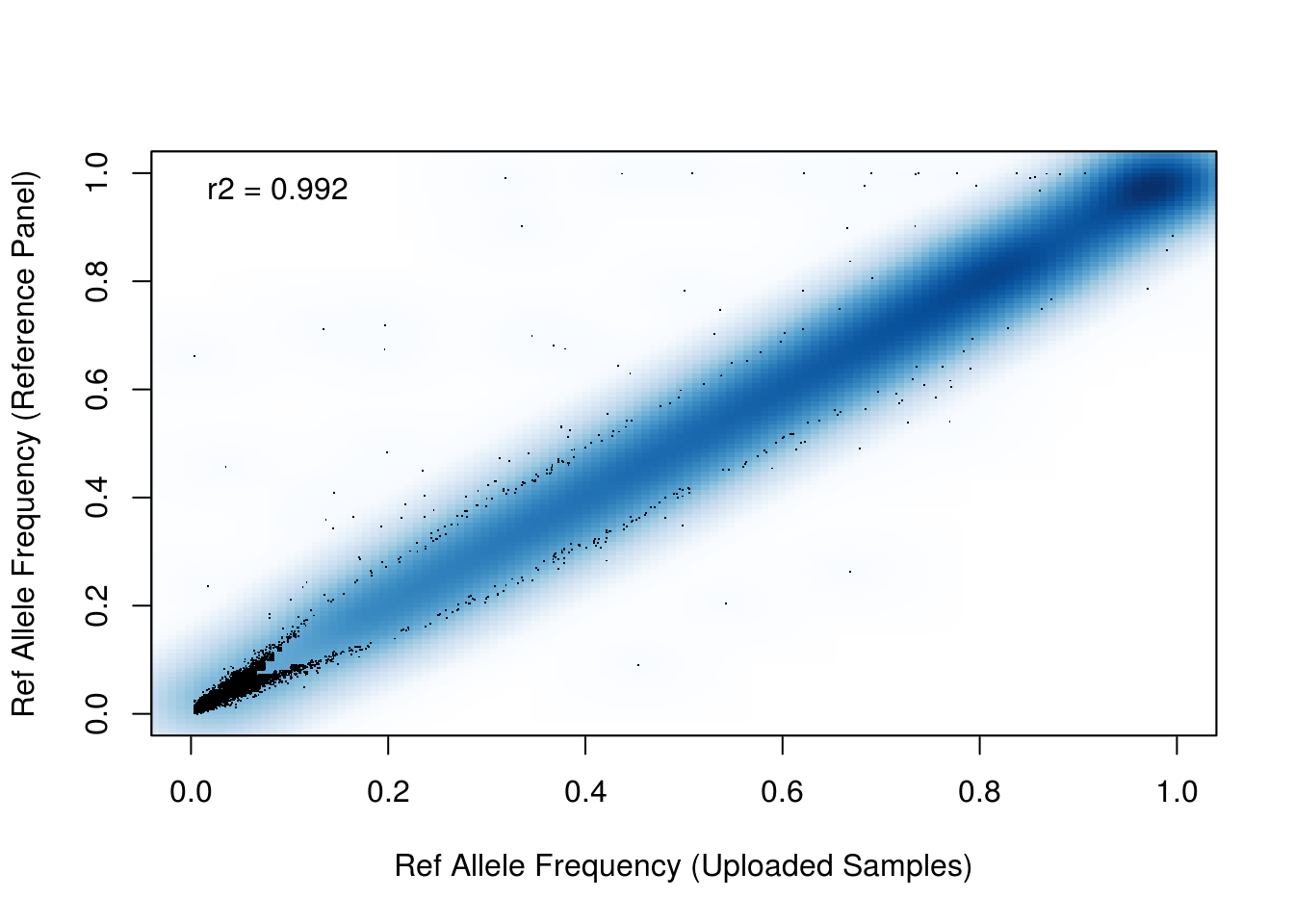


1. **Quality control after imputation.**

Poor quality SNPs were excluded: multi-allelic SNPs, SNPs with a minor allele frequency (MAF)<0.0016 or INFO<0.3, and strand ambiguous AT/CG SNPs. The VCF dosage files were converted into PLINK format dosage files. Finally, 10,356,437 SNPs and 3,104 individuals remained. For hard call (best guess) genotypes, an additional SNP QC (INFO>0.8 and HWE>10-6) was performed, resulting in 6,436,459 SNPs.

1. **Principle components analyses (PCA)**

The principal components (PCs) analyses within NEMESIS-2 samples, and NEMESIS-2 samples along with Hapmap3([cited 2018 Sep 16] ) populations were conducted by EIGENSTRAT(Price *et al.*, 2006). A strict selection for SNPs with overlap with Hapmap3 SNPs was conducted: 1. MAF>0.05 and HWE>0.001; 2. Removal of 24 long LD regions (**Table 1**); 3. LD pruning with an R^2^ of 0.5; which resulted in 40,732 best quality genotyped SNPs used to calculate genetic PCs.

PCs were firstly calculated with HapMap3 population to exclude European ethnic outliers: exceeding 5 times the standard deviation of Utah residents with Northern and Western European ancestry from the CEPH collection (CEU) and Toscani in Italia (TSI) populations for the first 2 PCs (**Figure 2)**. The first 2 PCs explained > 84.3% of the total. In total, 141 individuals were excluded from this dataset. Secondly, another PCA was conducted using the same SNPs (n=40,732) but calculated only in NEMESIS-2. The first 2 PCs explained >30% of the total. Another 13 individuals were considered as ethnic outliers by the first 2 PCs exceeding 5 times the standard deviation in the NEMESIS-2 sample. In the end, 3,104 individuals remained. Compared with the self-report ethnic information, 93.75% of our ethnic-QCed samples are self-reported as “Dutch”. We think some people may have understood the question as: “where were you born?”, since non-former colony countries have 0 deviations from genetic QC, while former colonies have substantial deviations. In total, 154 samples were identified as European ethnic outliers and excluded. After post-imputation quality control steps, PCA was conducted using the same SNPs (n=40,732) within NEMESIS-2 samples (n=3,104). These PCs were then used as covariates in analyses to correct for population stratification.

## Figure 2. First 2PCs OF NEMESIS-2 samples with Hapmap 3 before exclusion of ethnic outliers

## Supplemental Table 1: The complex-LD regions (build GRCh37) removed for PCA analysis.

| Chromosome | Base pair start | Base pair end |
| --- | --- | --- |
| 6 | 25392021 | 33392022 |
| 8 | 111930824 | 114930824 |
| 11 | 46043424 | 57243424 |
| 1 | 48287980 | 52287979 |
| 2 | 86088342 | 101041482 |
| 2 | 134666268 | 138166268 |
| 2 | 183174494 | 190174494 |
| 3 | 47524996 | 50024996 |
| 3 | 83417310 | 86917310 |
| 3 | 88917310 | 96017310 |
| 5 | 44464243 | 50464243 |
| 5 | 97972100 | 100472101 |
| 5 | 128972101 | 131972101 |
| 5 | 135472101 | 138472101 |
| 6 | 56892041 | 63942041 |
| 6 | 139958307 | 142458307 |
| 7 | 55225791 | 66555850 |
| 8 | 7962590 | 11962591 |
| 8 | 42880843 | 49837447 |
| 10 | 36959994 | 43679994 |
| 11 | 87860352 | 90860352 |
| 12 | 33108733 | 41713733 |
| 12 | 111037280 | 113537280 |
| 20 | 32536339 | 35066586 |

## B. Training schizophrenia GWAS summary statistic processing and calculating polygenic risk score for schizophrenia

We used recent GWASs of schizophrenia (Pardinas *et al.*, 2018) for PRS calculations (Choi *et al.*, 2018). As a quality control for PRS calculation, the SNPs that overlapped between the GWASs summary statistics (training datasets) and our dataset were extracted. Then, insertions or deletions, ambiguous SNPs, SNPs with minor allele frequency (MAF) <0.01 and imputation quality (R^2^) < 0.8 in both training and target datasets were excluded. To account for complicated LD structure of SNPs in the genome, these SNPs were clumped in two rounds using PLINK 1.90b3z(Chang *et al.*, 2015) according to previously established methods (McLaughlin *et al.*, 2017; Schur *et al.*, 2019); round 1 with the default parameters (physical distance threshold 250kb and LD threshold (R^2^) 0.5); round 2 with a physical distance threshold of 5,000kb and LD threshold (R^2^) 0.2. Additionally, we excluded all SNPs in genomic regions with strong or complex LD structures (e.g. the MHC region on chromosome 6; **Table 1**). The odds ratios (ORs) were reported in the summary statistics, and were log-converted to beta values as effect sizes. Sample overlap between NEMESIS-2 data with schizophrenia GWAS cohort (PCG and CLOZUK cohorts) is unlikely since all samples belong to different cohorts. We constructed PRS based on schizophrenia risk alleles weighted by their schizophrenia increasing effect estimate using the Purcell et al. method (Purcell *et al.*, 2007; International Schizophrenia Consortium *et al.*, 2009), i.e. using PLINK’s score function. PRS was calculated for 3,104 samples (those remaining after QC). Informed by the PGC analyses, PRS for schizophrenia with a significance cut-off *P* < 0.05 was used in the analyses to achieve a balance between the number of false-positive and true-positive risk alleles(Wray *et al.*, 2014).

# RESULTS

## Supplemental Table 2: Comparison of frequencies of time-varying and fixed variables in analysis as a function of missing PRS data in the risk set

|  | PRS data (n=9,339) | Missing PRS data (n=9776) | Total (n=19,115) |
| --- | --- | --- | --- |
| Young age group | 0.16 | 0.16 | 0.16 |
| Female sex | 0.55 | 0.54 | 0.55 |
| Perceived status gap | 0.16 | 0.21 | 0.18 |
| Low social functioning | 0.31 | 0.34 | 0.33 |
| Any care | 0.12 | 0.12 | 0.12 |
| Mental health care | 0.06 | 0.07 | 0.06 |
| Antipsychotic use | 0.00 | 0.01 | 0.00 |
| Cannabis use | 0.02 | 0.02 | 0.02 |
| Childhood adversity | 0.18 | 0.18 | 0.18 |
| Life events | 0.47 | 0.47 | 0.47 |
| Hearing impairment | 0.03 | 0.02 | 0.03 |
| Urbanicity | 0.37 | 0.4 | 0.39 |
| Family history | 0.59 | 0.58 | 0.59 |
| JTC bias | 0.49 | 0.53 | 0.51 |
| Altered digit symbol | 0.28 | 0.3 | 0.29 |

## Supplemental Table 3: Pattern of time-varying and time-constant exposures for PE-only and PE+NPD incidence analysis

| Exposure | PE-only incidence analysis | | | PE+NPD incidence analysis | | |
| --- | --- | --- | --- | --- | --- | --- |
|  | Constant | Varying | Never missing | Constant | Varying | Never missing |
| Young age group | 4600 | 330 | 4930 | 4589 | 341 | 4930 |
| Female sex | 4930 | 0 | 4930 | 4930 | 0 | 4930 |
| Perceived status gap | 3871 | 1059 | 4930 | 3838 | 1092 | 4930 |
| Low social functioning | 3088 | 1842 | 4929 | 3013 | 1917 | 4928 |
| Any care | 4042 | 888 | 4930 | 4013 | 917 | 4930 |
| Mental health care | 4413 | 517 | 4930 | 4411 | 519 | 4930 |
| Antipsychotic use | 4905 | 25 | 4930 | 4907 | 23 | 4930 |
| Cannabis use | 4850 | 14 | 4700 | 4851 | 11 | 4698 |
| Childhood adversity | 4930 | 0 | 4930 | 4930 | 0 | 4930 |
| Life events | 2217 | 2713 | 4930 | 2125 | 2805 | 4930 |
| Ethnic minority | 4930 | 0 | 4930 | 4930 | 0 | 4930 |
| Hearing impairment | 4930 | 0 | 4930 | 4930 | 0 | 4930 |
| Urbanicity <16 yrs | 4926 | 0 | 4926 | 4926 | 0 | 4926 |
| Family history | 4930 | 0 | 4930 | 4930 | 0 | 4930 |
| PRS_75_ | 2412 | 0 | 2412 | 2412 | 0 | 2412 |
| JTC bias | 4293 | 0 | 4293 | 4293 | 0 | 4293 |
| Altered digit symbol | 3595 | 0 | 3595 | 3595 | 0 | 3595 |

## Supplemental Table 4: Person-year incidence rates for PE-only and PE+NPD as a function of absence (0) or presence (1) of binary risk factors

| Binary exposure |  | PE-only | | | | | PE+NPD | | | | |
| --- | --- | --- | --- | --- | --- | --- | --- | --- | --- | --- | --- |
|  |  | n | Observations | Failures | Time | Incidence | n | Observations | Failures | Time | Incidence |
|  |  |  |  |  |  |  |  |  |  |  |  |
| Young age group | 0 | 4425 | 11012 | 329 | 33215.7 | 0.99% | 4436 | 11254 | 114 | 33948.2 | 0.34% |
|  | 1 | 835 | 1547 | 66 | 4682.5 | 1.41% | 835 | 1577 | 28 | 4773.2 | 0.59% |
| Female sex | 0 | 2238 | 5705 | 154 | 17183.6 | 0.90% | 2238 | 5839 | 53 | 17588.1 | 0.30% |
|  | 1 | 2692 | 6854 | 241 | 20714.5 | 1.16% | 2692 | 6992 | 89 | 21133.2 | 0.42% |
| Perceived status gap | 0 | 4417 | 10170 | 287 | 30671.8 | 0.94% | 4428 | 10403 | 68 | 31378.0 | 0.22% |
|  | 1 | 1572 | 2389 | 108 | 7226.3 | 1.49% | 1594 | 2428 | 74 | 7343.3 | 1.01% |
| Low social functioning | 0 | 4112 | 8372 | 231 | 25255.2 | 0.91% | 4136 | 8559 | 34 | 25822.4 | 0.13% |
|  | 1 | 2660 | 4186 | 164 | 12639.8 | 1.30% | 2711 | 4270 | 107 | 12892.8 | 0.83% |
| Any care | 0 | 4699 | 11054 | 337 | 33335.8 | 1.01% | 4698 | 11338 | 50 | 34194.6 | 0.15% |
|  | 1 | 1119 | 1505 | 58 | 4562.3 | 1.27% | 1149 | 1493 | 92 | 4526.8 | 2.03% |
| Mental health care | 0 | 4812 | 11745 | 368 | 35427.1 | 1.04% | 4800 | 12030 | 82 | 36290.3 | 0.23% |
|  | 1 | 635 | 814 | 27 | 2471.0 | 1.09% | 649 | 801 | 60 | 2431.1 | 2.47% |
| Antipsychotic use | 0 | 4920 | 12507 | 391 | 37742.5 | 1.04% | 4920 | 12786 | 135 | 38587.0 | 0.35% |
|  | 1 | 35 | 52 | 4 | 155.6 | 2.57% | 33 | 45 | 7 | 134.3 | 5.21% |
| Cannabis use | 0 | 4840 | 12158 | 379 | 36687.1 | 1.03% | 4835 | 12432 | 121 | 37517.4 | 0.32% |
|  | 1 | 38 | 48 | 3 | 146.8 | 2.04% | 38 | 46 | 5 | 140.3 | 3.56% |
| Childhood adversity | 0 | 4039 | 10423 | 295 | 31435.8 | 0.94% | 4039 | 10659 | 79 | 32150.2 | 0.25% |
|  | 1 | 891 | 2136 | 100 | 6462.3 | 1.55% | 891 | 2172 | 63 | 6571.1 | 0.96% |
| Life events | 0 | 3981 | 6773 | 185 | 20443.1 | 0.90% | 4016 | 6897 | 47 | 20818.1 | 0.23% |
|  | 1 | 3662 | 5786 | 210 | 17455.0 | 1.20% | 3719 | 5934 | 95 | 17903.2 | 0.53% |
| Ethnic minority | 0 | 4584 | 11737 | 362 | 35407.4 | 1.02% | 4584 | 12003 | 122 | 36211.3 | 0.34% |
|  | 1 | 346 | 822 | 33 | 2490.7 | 1.32% | 346 | 828 | 20 | 2510.0 | 0.80% |
| Hearing impairment | 0 | 4788 | 12200 | 373 | 36817.1 | 1.01% | 4788 | 12448 | 134 | 37568.3 | 0.36% |
|  | 1 | 142 | 359 | 22 | 1081.0 | 2.04% | 142 | 383 | 8 | 1153.0 | 0.69% |
| Urbanicity <16 yrs | 0 | 3036 | 7704 | 258 | 23251.5 | 1.11% | 3036 | 7918 | 73 | 23901.5 | 0.31% |
|  | 1 | 1890 | 4843 | 137 | 14611.0 | 0.94% | 1890 | 4901 | 69 | 14784.2 | 0.47% |
| Family history | 0 | 2193 | 5586 | 116 | 16846.3 | 0.69% | 2193 | 5689 | 12 | 17157.5 | 0.07% |
|  | 1 | 2737 | 6973 | 279 | 21051.8 | 1.33% | 2737 | 7142 | 130 | 21563.8 | 0.60% |
| PRS_75_ | 0 | 1806 | 4709 | 151 | 14222.1 | 1.06% | 1806 | 4843 | 33 | 14623.8 | 0.23% |
|  | 1 | 606 | 1577 | 40 | 4763.3 | 0.84% | 606 | 1588 | 28 | 4797.5 | 0.58% |
| JTC bias | 0 | 2098 | 5830 | 172 | 17563.0 | 0.98% | 2098 | 5957 | 55 | 17946.0 | 0.31% |
|  | 1 | 2195 | 6055 | 195 | 18264.4 | 1.07% | 2195 | 6196 | 75 | 18692.5 | 0.40% |
| Altered digit symbol | 0 | 2559 | 7454 | 188 | 22451.0 | 0.84% | 2559 | 7610 | 54 | 22924.2 | 0.24% |
|  | 1 | 1036 | 2965 | 116 | 8930.3 | 1.30% | 1036 | 3043 | 45 | 9166.5 | 0.49% |

## **Supplemental Table 5:** Sensitivity analysis of differential associations of incident Psychotic Experiences (PE), alone (PE-only) and in the context of Non-Psychotic Disorder (PE+NPD), with demographic, clinical, aetiological and cognitive factors, with more exclusive risk set (all individuals with PE at baseline excluded)

| Binary exposure | PE-only | | | | PE+NPD | | | |
| --- | --- | --- | --- | --- | --- | --- | --- | --- |
|  | HR* | 95% CI | | p | HR | 95% CI | | p |
| Young age group | 1.45 | 1.06 | 1.99 | 0.019 | 2.40 | 1.48 | 3.88 | 0.000 |
| Female sex | 1.30 | 1.03 | 1.64 | 0.028 | 1.33 | 0.88 | 2.01 | 0.171 |
| Perceived status gap | 1.74 | 1.35 | 2.25 | 0.000 | 5.60 | 3.74 | 8.37 | 0.000 |
| Low social functioning | 1.49 | 1.18 | 1.88 | 0.001 | 6.84 | 4.25 | 11.02 | 0.000 |
| Any care | 1.22 | 0.87 | 1.70 | 0.252 | 10.85 | 7.24 | 16.25 | 0.000 |
| Mental health care | 0.92 | 0.56 | 1.50 | 0.726 | 10.92 | 7.27 | 16.42 | 0.000 |
| Antipsychotic use | 1.16 | 0.16 | 8.30 | 0.879 | 19.85 | 7.26 | 54.25 | 0.000 |
| Cannabis use | 1.98 | 0.49 | 7.97 | 0.335 | 14.60 | 5.34 | 39.92 | 0.000 |
| Childhood adversity | 1.41 | 1.07 | 1.87 | 0.016 | 3.22 | 2.13 | 4.86 | 0.000 |
| Life events | 1.20 | 0.96 | 1.51 | 0.115 | 2.47 | 1.61 | 3.79 | 0.000 |
| Ethnic minority | 1.29 | 0.85 | 1.95 | 0.237 | 2.08 | 1.14 | 3.81 | 0.017 |
| Hearing impairment | 2.25 | 1.40 | 3.63 | 0.001 | 2.17 | 0.95 | 4.97 | 0.066 |
| Urbanicity <16 yrs | 0.82 | 0.65 | 1.05 | 0.110 | 1.21 | 0.81 | 1.81 | 0.359 |
| Family history | 1.92 | 1.50 | 2.46 | 0.000 | 7.30 | 3.79 | 14.05 | 0.000 |
| PRS_75_ | 0.87 | 0.58 | 1.32 | 0.520 | 2.05 | 1.10 | 3.84 | 0.025 |
| JTC bias | 1.07 | 0.85 | 1.36 | 0.557 | 1.25 | 0.82 | 1.90 | 0.302 |
| Altered digit symbol | 1.60 | 1.23 | 2.10 | 0.001 | 1.94 | 1.21 | 3.12 | 0.006 |

HR = hazard ratio, 95% CI = 95% confidence interval

Young age group: aged 18-35 years; Perceived status gap: difference between actual and desired social position; Low social functioning: SF36 social functioning 75^th^ percentile cut-off; Any care: any informal, medical or mental health care for mental problems or addiction; Cannabis use: once per week or more in the period most frequent use; Childhood adversity: 80^th^ percentile cut-off continuous adversity score before age 16 years; Life events: at least one life event in the last year; Minority: Moroccan, Turkish, Surinamese, Antillean, Indonesian or other non-western ethnic group; Hearing impairment: T0 deafness or serious hearing impairment in the past 12 months; Urbanicity 2 highest levels of 5-level urbanicity classification before age 16 years; Family history: family history mental disorder; PRS_75_: schizophrenia polygenic risk score 75^th^ percentile cut-off; JTC: beads task decision 2 or less beads; Altered digit symbol: cut-off 75^th^ percentile continuous score.

# = zero cells

HR significantly greater in PE+NPD group compared to PE-only group, based on non-overlapping confidence intervals

HR significant in PE+NPD group but not in PE-only group

## **Supplemental Table 6:** Sensitivity analysis of differential associations of incident Psychotic Experiences (PE), alone (PE-only) and in the context of Non-Psychotic Disorder (PE+NPD), with continuous measures of exposure instead of dichotomization around the x^th^ percentile

| Binary exposure | PE-only | | | | PE+NPD | | | |
| --- | --- | --- | --- | --- | --- | --- | --- | --- |
|  | HR* | 95% CI | | p | HR | 95% CI | | p |
| Low social functioning | 1.40 | 1.15 | 1.71 | 0.001 | 6.21 | 4.22 | 9.14 | 0.000 |
| Childhood adversity | 1.24 | 1.14 | 1.35 | 0.000 | 1.61 | 1.47 | 1.77 | 0.000 |
| PRS | 0.99 | 0.95 | 1.02 | 0.393 | 1.14 | 1.07 | 1.21 | 0.000 |
| Digit symbol score | 0.93 | 0.90 | 0.96 | 0.000 | 0.89 | 0.83 | 0.94 | 0.000 |

HR = hazard ratio, 95% CI = 95% confidence interval

Low social functioning: SF36 social functioning; Childhood adversity: Continuous adversity score before age 16 years; PRS: Schizophrenia polygenic risk score; Digit symbol score: continuous score.

HR significantly greater in PE+NPD group compared to PE-only group, based on non-overlapping confidence intervals

## Sensitivity analysis in individuals aged 18-35 years at baseline

Sensitivity analyses restricted to individuals aged 18-35 at baseline (n=1439, 26%) revealed a very similar pattern of results (Fig. 2).

**Figure 2.** Hazard ratio (HR) effect sizes of binary clinical, demographic, etiological and cognitive factors in PE-only group relative to effect sizes of PE+NPD group (set at 100%, grey line), sample confined to those aged 18-35 years at baseline

*Perceived status gap: difference between actual and desired social position; Low social functioning: SF36 social functioning 75th percentile cut-off; Any care: any informal, medical or mental health care for mental problems or addiction; Cannabis use: once per week or more in the period most frequent use; Childhood adversity: 80th percentile cut-off continuous adversity score before age 16 years; Life events: at least one life event in the last year; Minority: Moroccan, Turkish, Surinamese, Antillean, Indonesian or other non-western ethnic group; Hearing impairment: T0 deafness or serious hearing impairment in the past 12 months; Urbanicity 2 highest levels of 5-level urbanicity classification before age 16 years; Family history: family history mental disorder; PRS_75_: schizophrenia polygenic risk score 75th percentile cut-off; JTC: beads task decision 2 or less beads; Altered digit symbol: cut-off 75th percentile continuous score.*

# DISCUSSION

## Supplemental Table 7. Sensitivity analyses of association between PRS, modelled as continuous quartile-group variable, and incidence of PE+NPD phenotype, showing dose-response relationship and significant linear trend.

| PRS Quartile groups |  |  |  |  |  |
| --- | --- | --- | --- | --- | --- |
|  | PE+NPD | | | | |
|  |  | HR | 95% CI | | p |
| 1* | 1* |  |  |  |  |
| 2 | 2 | 1.15 | 0.42 | 3.18 | 0.783 |
| 3 | 3 | 2.60 | 1.09 | 6.24 | 0.032 |
| 4 | 4 | 4.10 | 1.79 | 9.39 | 0.001 |
|  |  |  |  |  |  |
| HR linear trend |  | 1.69 | 1.31 | 2.16 | 0.000 |

HR = hazard ratio

95% CI = 95% confidence interval

p = p-value

1* = reference category

# REFERENCES

([cited 2018 Sep 16] ) HapMap 3 [Internet] Available from: <https://www.sanger.ac.uk/resources/downloads/human/hapmap3.html>.

**Adler NE, Epel ES, Castellazzo G and Ickovics JR**. (2000) Relationship of subjective and objective social status with psychological and physiological functioning: preliminary data in healthy white women. *Health Psychology* **19**: 586-592.

**Alonso J, Angermeyer MC, Bernert S, Bruffaerts R, Brugha TS, Bryson H, de Girolamo G, Graaf R, Demyttenaere K, Gasquet I, Haro JM, Katz SJ, Kessler RC, Kovess V, Lepine JP, Ormel J, Polidori G, Russo LJ, Vilagut G, Almansa J, Arbabzadeh-Bouchez S, Autonell J, Bernal M, Buist-Bouwman MA, Codony M, Domingo-Salvany A, Ferrer M, Joo SS, Martinez-Alonso M, Matschinger H, Mazzi F, Morgan Z, Morosini P, Palacin C, Romera B, Taub N, Vollebergh WA and EsemeD/Mhedea Investigators - European Study of the Epidemiology of Mental Disorders Project**. (2004) Sampling and methods of the European Study of the Epidemiology of Mental Disorders (ESEMeD) project. *Acta Psychiatrica Scandinavica Supplementum*: 8-20.

**Bak M, Delespaul P, Hanssen M, de Graaf R, Vollebergh W and van Os J**. (2003) How false are "false" positive psychotic symptoms? *Schizophrenia Research* **62**: 187-189.

**Bijl RV and Ravelli A**. (2000) Psychiatric morbidity, service use, and need for care in the general population: results of The Netherlands Mental Health Survey and Incidence Study. *American journal of public health* **90**: 602.

**Bijl RV, Ravelli A and van Zessen G**. (1998) Prevalence of psychiatric disorder in the general population: results of the Netherlands Mental Health Survey and Incidence Study (NEMESIS). *Social Psychiatry and Psychiatric Epidemiology* **33**: 587-595.

**Brugha T, Bebbington P, Tennant C and Hurry J**. (1985) The List of Threatening Experiences: a subset of 12 life event categories with considerable long-term contextual threat. *Psychol Med* **15**: 189-194.

**Chang CC, Chow CC, Tellier LC, Vattikuti S, Purcell SM and Lee JJ**. (2015) Second-generation PLINK: rising to the challenge of larger and richer datasets. *Gigascience* **4**: 7.

**Choi SW, Heng Mak TS and O’Reilly PF**. (2018) A guide to performing Polygenic Risk Score analyses. *bioRxiv*.

**de Graaf R, ten Have M, Burger H and Buist-Bouwman M**. (2008) Mental disorders and service use in the Netherlands. Results from the European Study of the Epidemiology of Mental Disorders (ESEMeD) In: Ustun T and Kessler R (eds) *The WHO World Mental Health Surveys: Global Perspectives on the Epidemiology of Mental Disorders.* New York: Cambridge University Press, 388–405.

**de Graaf R, Ten Have M and van Dorsselaer S**. (2010) The Netherlands Mental Health Survey and Incidence Study-2 (NEMESIS-2): design and methods. *Int J Methods Psychiatr Res* **19**: 125-141.

**de Graaf R, ten Have M, van Gool C and van Dorsselaer S**. (2012) Prevalence of mental disorders and trends from 1996 to 2009. Results from the Netherlands Mental Health Survey and Incidence Study-2. *Social Psychiatry and Psychiatric Epidemiology* **47**: 203-213.

**de Graaf R, van Dorsselaer S, Tuithof M and ten Have M**. (2018) Sociodemographic and psychiatric predictors of attrition in the third follow-up of the Netherlands Mental Health Survey and Incidence Study-2 (NEMESIS2). Utrecht: Trimbos Institute.

**EUGEI investigators**. (2014) Identifying gene-environment interactions in schizophrenia: contemporary challenges for integrated, large-scale investigations. *Schizophr Bull* **40**: 729-736.

**Guloksuz S, van Nierop M, Lieb R, van Winkel R, Wittchen HU and van Os J**. (2015) Evidence that the presence of psychosis in non-psychotic disorder is environment-dependent and mediated by severity of non-psychotic psychopathology. *Psychological Medicine* **45**: 2389-2401.

**Haro JM, Arbabzadeh-Bouchez S, Brugha TS, de Girolamo G, Guyer ME, Jin R, Lepine JP, Mazzi F, Reneses B, Vilagut G, Sampson NA and Kessler RC**. (2006) Concordance of the Composite International Diagnostic Interview Version 3.0 (CIDI 3.0) with standardized clinical assessments in the WHO World Mental Health surveys. *Int J Methods Psychiatr Res* **15**: 167-180.

**Heins M, Simons C, Lataster T, Pfeifer S, Versmissen D, Lardinois M, Marcelis M, Delespaul P, Krabbendam L, van Os J and Myin-Germeys I**. (2011) Childhood trauma and psychosis: a case-control and case-sibling comparison across different levels of genetic liability, psychopathology, and type of trauma. *American Journal of Psychiatry* **168**: 1286-1294.

**Helzer JE, Robins LN, McEvoy LT, Spitznagel EL, Stoltzman RK, Farmer A and Brockington IF**. (1985) A comparison of clinical and diagnostic interview schedule diagnoses. Physician reexamination of lay-interviewed cases in the general population. *Archives of General Psychiatry* **42**: 657-666.

**International Schizophrenia Consortium, Purcell SM, Wray NR, Stone JL, Visscher PM, O'Donovan MC, Sullivan PF and Sklar P**. (2009) Common polygenic variation contributes to risk of schizophrenia and bipolar disorder. *Nature* **460**: 748-752.

**McLaughlin RL, Schijven D, van Rheenen W, van Eijk KR, O'Brien M, Kahn RS, Ophoff RA, Goris A, Bradley DG, Al-Chalabi A, van den Berg LH, Luykx JJ, Hardiman O, Veldink JH, Project Min EGC and Schizophrenia Working Group of the Psychiatric Genomics C**. (2017) Genetic correlation between amyotrophic lateral sclerosis and schizophrenia. *Nature Communications* **8**: 14774.

**Menon M, Pomarol-Clotet E, McCarthy R and McKenna P**. (2002) Probabilistic reasoning bias is a function of having schizophrenia, not of being deluded. *Schizophrenia Research.* ELSEVIER SCIENCE BV PO BOX 211, 1000 AE AMSTERDAM, NETHERLANDS, 133-133.

**Moritz S and Woodward TS**. (2005) Jumping to conclusions in delusional and non‐delusional schizophrenic patients. *British Journal of Clinical Psychology* **44**: 193-207.

**Pardinas AF, Holmans P, Pocklington AJ, Escott-Price V, Ripke S, Carrera N, Legge SE, Bishop S, Cameron D, Hamshere ML, Han J, Hubbard L, Lynham A, Mantripragada K, Rees E, MacCabe JH, McCarroll SA, Baune BT, Breen G, Byrne EM, Dannlowski U, Eley TC, Hayward C, Martin NG, McIntosh AM, Plomin R, Porteous DJ, Wray NR, Caballero A, Geschwind DH, Huckins LM, Ruderfer DM, Santiago E, Sklar P, Stahl EA, Won H, Agerbo E, Als TD, Andreassen OA, Baekvad-Hansen M, Mortensen PB, Pedersen CB, Borglum AD, Bybjerg-Grauholm J, Djurovic S, Durmishi N, Pedersen MG, Golimbet V, Grove J, Hougaard DM, Mattheisen M, Molden E, Mors O, Nordentoft M, Pejovic-Milovancevic M, Sigurdsson E, Silagadze T, Hansen CS, Stefansson K, Stefansson H, Steinberg S, Tosato S, Werge T, Consortium G, Consortium C, Collier DA, Rujescu D, Kirov G, Owen MJ, O'Donovan MC and Walters JTR**. (2018) Common schizophrenia alleles are enriched in mutation-intolerant genes and in regions under strong background selection. *Nature Genetics* **50**: 381-389.

**Phillips LD and Edwards W**. (1966) Conservatism in a simple probability inference task. *Journal of experimental psychology* **72**: 346.

**Price AL, Patterson NJ, Plenge RM, Weinblatt ME, Shadick NA and Reich D**. (2006) Principal components analysis corrects for stratification in genome-wide association studies. *Nature Genetics* **38**: 904-909.

**Pries LK, Guloksuz S, Ten Have M, de Graaf R, van Dorsselaer S, Gunther N, Rauschenberg C, Reininghaus U, Radhakrishnan R, Bak M, Rutten BPF and van Os J**. (2018) Evidence That Environmental and Familial Risks for Psychosis Additively Impact a Multidimensional Subthreshold Psychosis Syndrome. *Schizophrenia Bulletin* **44**: 710-719.

**Purcell S, Neale B, Todd-Brown K, Thomas L, Ferreira MA, Bender D, Maller J, Sklar P, de Bakker PI, Daly MJ and Sham PC**. (2007) PLINK: a tool set for whole-genome association and population-based linkage analyses. *American Journal of Human Genetics* **81**: 559-575.

**Radhakrishnan R, Guloksuz S, Ten Have M, de Graaf R, van Dorsselaer S, Gunther N, Rauschenberg C, Reininghaus U, Pries LK, Bak M and van Os J**. (2019) Interaction between environmental and familial affective risk impacts psychosis admixture in states of affective dysregulation. *Psychological Medicine* **49**: 1879-1889.

**Rauschenberg C, Reininghaus U, Ten Have M, de Graaf R, van Dorsselaer S, Simons CJP, Gunther N, Henquet C, Pries LK, Guloksuz S, Bak M and van Os J**. (2020) The jumping to conclusions reasoning bias as a cognitive factor contributing to psychosis progression and persistence: findings from NEMESIS-2. *Psychological Medicine*: 1-8.

**Reed V, Gander F, Pfister H, Steiger A, Sonntag H, Trenkwalder C, Sonntag A, Hundt W and Wittchen H-U**. (1998) To what degree does the Composite International Diagnostic Interview (CIDI) correctly identify DSM-IV disorders? Testing validity issues in a clinical sample. *International Journal of Methods in Psychiatric Research* **7**: 142-155.

**Reininghaus U, Rauschenberg C, Ten Have M, de Graaf R, van Dorsselaer S, Simons CJP, Gunther N, Pries LK, Guloksuz S, Radhakrishnan R, Bak M and van Os J**. (2019) Reasoning bias, working memory performance and a transdiagnostic phenotype of affective disturbances and psychotic experiences in the general population. *Psychological Medicine* **49**: 1799-1809.

**Schur RR, Schijven D, Boks MP, Rutten BPF, Stein MB, Veldink JH, Joels M, Geuze E, Vermetten E, Luykx JJ and Vinkers CH**. (2019) The effect of genetic vulnerability and military deployment on the development of post-traumatic stress disorder and depressive symptoms. *Eur Neuropsychopharmacol* **29**: 405-415.

**Smeets F, Lataster T, van Winkel R, de Graaf R, Ten Have M and van Os J**. (2013) Testing the hypothesis that psychotic illness begins when subthreshold hallucinations combine with delusional ideation. *Acta Psychiatrica Scandinavica* **127**: 34-47.

**Stewart AL, Hays RD and Ware JE, Jr.** (1988) The MOS short-form general health survey. Reliability and validity in a patient population. *Medical Care* **26**: 724-735.

**Ten Have M, Nuyen J, Beekman A and de Graaf R**. (2013) Common mental disorder severity and its association with treatment contact and treatment intensity for mental health problems. *Psychological Medicine* **43**: 2203-2213.

**van Dam DS, van Nierop M, Viechtbauer W, Velthorst E, van Winkel R, Genetic R, Outcome of Psychosis i, Bruggeman R, Cahn W, de Haan L, Kahn RS, Meijer CJ, Myin-Germeys I, van Os J and Wiersma D**. (2015) Childhood abuse and neglect in relation to the presence and persistence of psychotic and depressive symptomatology. *Psychological Medicine* **45**: 1363-1377.

**van der Steen Y, Myin-Germeys I, van Nierop M, Ten Have M, de Graaf R, van Dorsselaer S, van Os J and van Winkel R**. (2019) 'False-positive' self-reported psychotic experiences in the general population: an investigation of outcome, predictive factors and clinical relevance. *Epidemiol Psychiatr Sci* **28**: 532-543.

**van Nierop M, van Os J, Gunther N, Myin-Germeys I, de Graaf R, ten Have M, van Dorsselaer S, Bak M and van Winkel R**. (2012) Phenotypically continuous with clinical psychosis, discontinuous in need for care: evidence for an extended psychosis phenotype. *Schizophrenia Bulletin* **38**: 231-238.

**Van Os J, Hanssen M, Bijl R and Ravelli A**. (2000) Straus (1969) revisited: A psychosis continuum in the general population? *Schizophrenia Research* **45**: 11-20.

**van Os J, Marsman A, van Dam D, Simons CJ and Investigators G**. (2017) Evidence That the Impact of Childhood Trauma on IQ Is Substantial in Controls, Moderate in Siblings, and Absent in Patients With Psychotic Disorder. *Schizophrenia Bulletin* **43**: 316-324.

**van Rossum I, Dominguez MD, Lieb R, Wittchen HU and van Os J**. (2011) Affective dysregulation and reality distortion: a 10-year prospective study of their association and clinical relevance. *Schizophrenia Bulletin* **37**: 561-571.

**Ware JE, Jr. and Sherbourne CD**. (1992) The MOS 36-item short-form health survey (SF-36). I. Conceptual framework and item selection. *Medical Care* **30**: 473-483.

**Wechsler D**. (1997) *WAIS-III: Wechsler Adult Intelligence Scale (3rd ed.) Administration and Scoring Manual.,* San Antonio, TX: Psychological Corporation.

**Wittchen HU**. (1994) Reliability and validity studies of the WHO--Composite International Diagnostic Interview (CIDI): a critical review. *Journal of Psychiatric Research* **28**: 57-84.

**World Health Organisation**. (1990) *Composite International Diagnostic Interview (CIDI) Version 1.0,* Geneva: World Health Organisation.

**Wray NR, Lee SH, Mehta D, Vinkhuyzen AAE, Dudbridge F and Middeldorp CM**. (2014) Research Review: Polygenic methods and their application to psychiatric traits. *Journal of Child Psychology and Psychiatry* **55**: 1068-1087.
